# Rheology, physicochemical properties, and microstructure of fish gelatin emulsion gel modified by γ-polyglutamic acid

**DOI:** 10.3389/fnut.2024.1343394

**Published:** 2024-03-20

**Authors:** Huan Xie, Xiao-Mei Sha, Ping Yuan, Jia-Le Li, Zi-Zi Hu, Zong-Cai Tu

**Affiliations:** ^1^National R&D Center for Freshwater Fish Processing, College of Chemistry and Chemical Engineering & College of Life Science, Jiangxi Normal University, Nanchang, China; ^2^Jiangxi Deshang Pharmaceutical Co., Ltd., Yichun, Jiangxi, China; ^3^State Key Laboratory of Food Science and Resources, Nanchang University, Nanchang, China

**Keywords:** fish gelatin, γ-polyglutamic acid, emulsion gel, rheology, physicochemical properties

## Abstract

In this work, the effect of the addition of γ-polyglutamic acid (γ-PGA) on the rheology, physicochemical properties, and microstructure of fish gelatin (FG) emulsion gel was investigated. Samples of the emulsion gel were evaluated for rheological behavior and stability prior to gelation. The mechanical properties and water-holding capacity (WHC) of the emulsion were determined after gelation. The microstructure of the emulsion gel was further examined using confocal laser scanning microscopy (CLSM). The results indicated a gradual increase in the apparent viscosity and gelation temperature of the emulsion at a higher concentration of γ-PGA. Additionally, frequency scan results revealed that on the addition of γ-PGA, FG emulsion exhibited a stronger structure. The emulsion containing 0.1% γ-PGA exhibited higher stability than that of the control samples. The WHC and gel strength of the emulsion gel increased on increasing the γ-PGA concentration. CLSM images showed that the addition of γ-PGA modified the structure of the emulsion gel, and the droplets containing 0.1% γ-PGA were evenly distributed. Moreover, γ-PGA could regulate the droplet size of the FG emulsion and its size distribution. These findings suggest that the viscoelasticity and structure of FG emulsion gels could be regulated by adjusting the γ-PGA concentration. The γ-PGA-modified FG emulsion gel also exhibited improved rheology and physicochemical properties. The results showed that γ-PGA-modified FG emulsion gel may find potential applications in food, medicine, cosmetics, and other industries.

## 1 Introduction

Emulsions, widely used in the food, pharmaceutical, and cosmetic industries, are thermodynamically unstable systems that tend to break down over time (1). Emulsion gels, which are soft solid materials, are basically emulsions with a gel network structure and stable mechanical properties (2). They are a type of complex colloid material consisting of proteins, polysaccharides, and other macromolecules (3). Emulsion gels combine the advantages of an emulsion and a gel, may provide better physical stability, and have more controllable structures, higher water-holding capacity, higher emulsion stability, and intestinal drug release properties compared with the traditional emulsions (4–6). In the liquid form, emulsion gels usually have higher viscoelasticity than conventional emulsions (4). In the solid form, they exhibit certain mechanical properties. Emulsion gels are prepared by various methods, such as heat treatment, cold curing, enzyme treatment, acidification, and ion addition (7–9). As structuring oils, emulsion gels have the potential to act as fat substitutes. They can replace fat in cookies, chocolate, sausage, and yogurt (10–12).

Emulsion gels are formed when the continuous phase of an emulsion undergoes gelation. Certain hydrocolloids, including carrageenan, whey protein isolate, and inulin, possess remarkable gelling and stabilizing properties, making them ideal gelling agents (13–15). Gelatin is a natural biological macromolecule derived from animal collagen, extracted mainly from pig, cow, or fish (16). Because of its functional properties such as good emulsification, biodegradability, and biocompatibility, gelatin is commonly used as a gelling agent, stabilizer, emulsifier, and thickener (16–18). FG is an environmentally friendly fish by-product. It can be used as an emulsifier and is capable of forming a gel matrix in emulsion gel systems. FG/sodium alginate emulsion gel can encapsulate probiotics (19). However, some of its properties are weak compared to other materials, leading to its poor stability and gelling properties. Therefore, various modifications have been made to improve its properties. FG–gum arabic interaction led to an increase in the stability of the emulsion gel (20). Pectin improved the physical stability and rheological properties of FG emulsions (21). TGase-modified FG increased the creaming stability of fish oil-loaded emulsions (22). Microwave processing technology enhanced the gel strength and emulsion stability index of FG (23).

One of the materials that can be employed to modify the properties of FG is γ-polyglutamic acid (γ-PGA). γ-PGA, also known as natrium gum, is an anionic polymer prepared from d-glutamic acid or l-glutamic acid, bonded by α-amino and γ-carboxyl groups, forming γ-amide bonds. γ-PGA exhibits good biocompatibility and good water solubility and is also degradable, edible, non-toxic, and moisturizing (24). Its conjugation and moisturizing properties have made it a promising biopolymer for versatile uses in the food industry, cosmetics, wastewater treatment, and biomedical product applications (25). Microgel-encapsulated probiotics prepared using γ-PGA have been shown to improve symptoms in mice with colitis. Double-network hydrogel based on γ-PGA and gelatin accelerated the wound healing process (26). Our previous studies have shown that γ-PGA can improve the gelling properties and rheological properties of FG. Among the composite emulsion gels, the polysaccharide–protein emulsion gel has attracted relatively more research attention, but the effect of γ-PGA in an emulsion gel system has not been studied (27). Whether γ-PGA can improve the gelling properties and stability of FG emulsion gel is worth an in-depth study.

In this work, we used corn oil as the oil phase and FG in combination with various concentrations of γ-PGA as the aqueous phase to develop a composite emulsion gel system. The rheological properties and stability were determined when the emulsion was in the liquid state. The mechanical properties and water-holding capacity (WHC) were evaluated when the emulsion became a gel. The microscopic morphologies of the FG emulsion gel were analyzed by confocal laser scanning microscopy (CLSM). By modifying the FG emulsion gel with γ-PGA, its application range can be expanded.

## 2 Materials and methods

### 2.1 Materials

FG (Type A gelatin, 260–270 Bloom) from Tilapia skin was purchased from Ji Li Ding Marine Biological Technology Co., Ltd. (Suzhou, China). γ-PGA (700 KDa) was purchased from Zelang Biotechnology Co., Ltd. (Shaanxi, China). Corn oil was obtained from Yi Haikerry Grain and Oil Food Company (Nanchang, China). All other reagents used were of analytical reagent grade.

### 2.2 Preparation of FG–γ-PGA emulsion gel

The FG–γ-PGA emulsion gel was prepared following Ding et al. (28) with appropriate modifications. FG was dissolved in ultrapure water to prepare a 2% (w/v) solution. Varying concentrations of γ-PGA solution were added to the FG solution: 0% (control), 0.02%, 0.04%, 0.06%, 0.08%, and 0.1% (w/v). An equal volume of corn oil was added to the FG solution. The FG–γ-PGA emulsion gel was formed after mechanically shearing the prepared solution for 3 min through a high-shear mixer (ULTRA TURRAX homogenizer, T25 digital, IKA, Staufen, Germany) at a homogenization speed of 13,000 rpm.

### 2.3 Rheological properties

The rheological properties of FG–γ-PGA emulsion were determined according to the method of Hu et al. (29) with some modifications. Measurements were recorded using a shear rate-controlled rheometer with 50 mm stainless steel parallel plates (MCR302 rheometer, Anton Paar, Austria). Continuous shear tests were performed over the shear rates of 0.01–100 s^−1^ at 25°C. A gap of 0.1 mm was fitted to the viscosity data of the emulsion. Frequency tests were performed on the gelled colloids with a strain of 0.5% (the linear viscoelastic interval is determined by amplitude scanning, and 0.5% strain was selected) at a frequency of 0.1–100 rad/s at 25°C.

The following measurements were performed using a rheometer with a 27 mL concentric cylinder. Temperature scan tests were performed to determine the energy storage modulus (G') and loss modulus (G”). Meanwhile, the data were recorded throughout the gelation process in the temperature range of about 5°C to 50°C. For each measurement, a fresh emulsion was added and the parameters used for this test were 0.5% strain and temperature increasing at 3°C/min.

### 2.4 Stability experiments

#### 2.4.1 Storage stability measurement

Refer to the method of Ding et al. (28). The prepared emulsions were placed in separate glass bottles and stored at room temperature. Pictures were taken at regular intervals and the morphology of the emulsion morphology was observed.

#### 2.4.2 Freeze–thaw stability measurement

Following the method of Liu et al. (30) with some modifications, the prepared emulsions were placed in a refrigerator at −20°C and frozen for about 20 h each time. After that, samples were furtherly thawed for about 4 h at 20°C. The cycle was repeated six times and photograph was taken each time.

### 2.5 Measurement of gelling properties

The emulsion gel was characterized using a texture analyzer (TA-XT-Plus Texture Analyzer, Stable Microsystems Ltd, Surrey, UK) equipped with a cylindrical measuring probe (P 0.5R) according to the method of Hu et al. (29) with appropriate modifications. A 19 mL sample was placed in a 25 mL beaker at 4°C for 16–18 h and the instrument was then calibrated (force and height calibration) prior to testing. The pre-test speed of the probe was 1.5 mm/s. The test speed was 1 mm/s. The post-test speed was 1 mm/s, and the trigger force was 2 g. The software installed in the instrument analyzed the resulting force–time curve and calculated the textural properties of the test sample, such as hardness and masticatory properties.

### 2.6 Measurement of water-holding capacity

According to the method of Yang et al. (31) with appropriate modifications, 20 g of sample was centrifuged in a 50 mL centrifuge tube at 8000 rpm for 30 min at 4°C. After centrifugation, the released water was collected and weighed. WHC was then calculated using the following formula: WHC = (W_t_ – W_f_)/ W_t_^*^ 100%. Here, W_t_ is the total mass of water in the original sample and W_f_ is the mass of water released after centrifugation.

### 2.7 Characterization of droplets

#### 2.7.1 Confocal laser scanning microscopy and optical measurement

The morphology of the emulsion could be observed according to the method of Chen et al. (32) with appropriate modifications. For liquid emulsion, 40 μL of fluorescent dye (0.02% Nile Red and 0.1% Nile Blue) was added to 1 mL of emulsion. The emulsion was vortexed for 1 min and incubated for 4 min in the dark. Then, 5 μL of the emulsion was added to a glass microslide and a square coverslip was placed on top of the emulsion. The microstructure of the emulsion under bright field and the microstructure of the emulsion under fluorescence excitation were observed simultaneously.

A confocal laser scanning microscope (TCS SP8 SR, Leica, Wetzlar, Germany) with a 40 × objective was used to observe these samples. The inner oil phase and outer water phase were stained using Nile Red and Nile Blue A, respectively. A 488-nm laser was applied to excite the Nile Red dye and 633-nm laser was applied to excite the Nile Blue dye (32, 33). After laser excitation, the emulsion sample was placed on a microscope slide and observed using CLSM. Three parallel specimens were observed with a scanning frequency of 200 Hz and a scanning density of 1024 × 1024 pixels. The images were acquired and analyzed by Leica Application Suite X software.

#### 2.7.2 Particle size measurement

The particle size of the different emulsions were measured using a laser particle size analyzer (NanoBrook-90PlusPALS; Brookhaven Instruments, Ltd., Holtsville, NY, USA) following the method described by Wang et al. (34). The fresh samples were diluted with deionized water to form a clear and transparent solution and then measured immediately.

### 2.8 Statistical analysis

All the experiments were carried out in triplicate, and results were presented as means and standard deviations. Data analysis was carried out by one-way ANOVA using SPSS software (Version 19.0), and values of *p* < 0.05 were considered statistically significant.

## 3 Results and discussion

### 3.1 Rheological characterization

#### 3.1.1 Apparent viscosity

**Figure 1:**
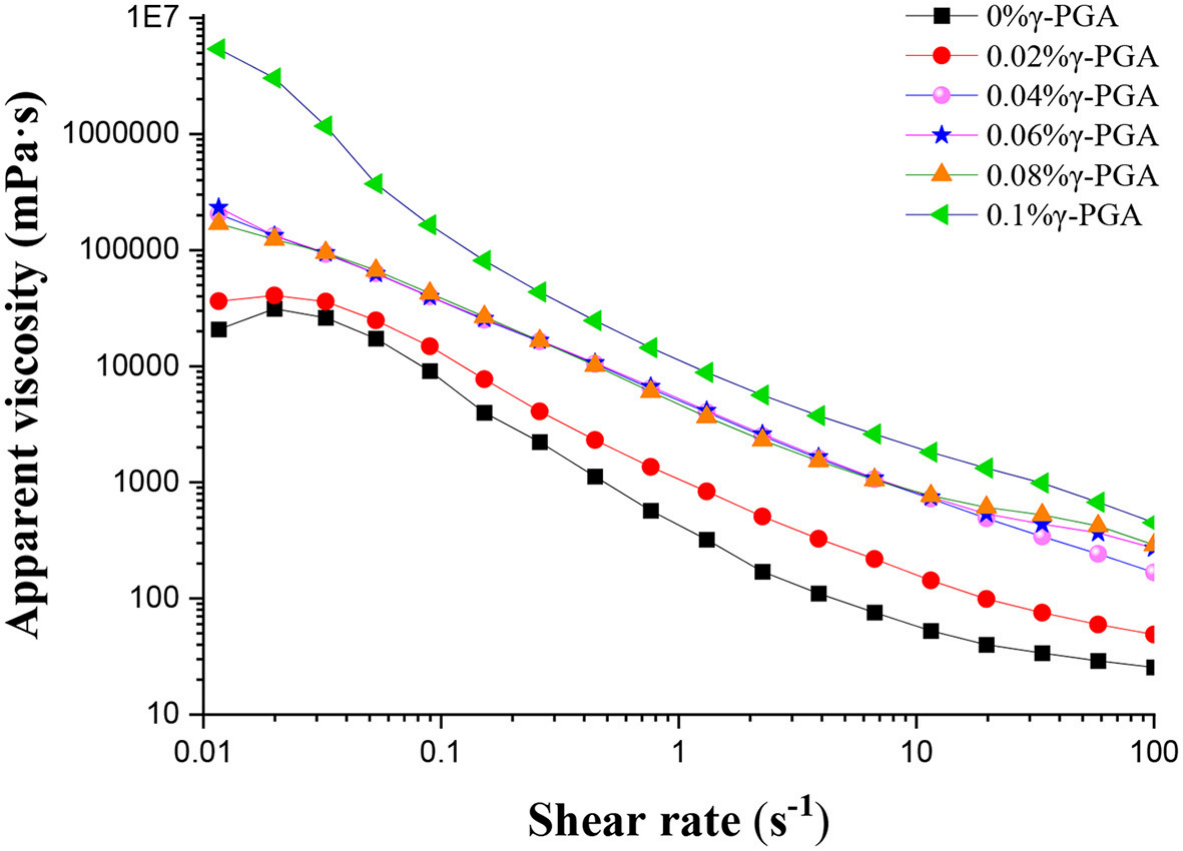
The apparent viscosity of FG emulsion modified by γ-PGA with different concentrations (0, 0.02, 0.04, 0.06, 0.08, 0.1% from top to bottom, respectively).

As depicted in Figure 1, the apparent viscosity of FG emulsion decreased with an increase in shear rate, irrespective of the addition of γ-PGA. The lowest apparent viscosity value was observed when the shear rate reached 100 s^−1^. The variation in viscosity, which was in line with the shear-thinning properties, is commonly observed in pseudoplastic fluids. The addition of xanthan gum to soy protein isolate emulsion showed pseudoplastic fluid characteristics (35). The whey protein isolate *Mesona chinensis* polysaccharide emulsions exhibited shear-thinning properties (36). The incorporation of fiber polysaccharide–protein extracted from *Haematococcus pluvialis* residues (FPHRs) also resulted in the presence of shear-thinning behavior in all emulsion gel samples. (37). The shear-thinning behavior of the emulsion might be related to the non-Newtonian behavior of the continuous phase (38). Under static or low flow rate conditions, the tightly entangled liquid exhibited a higher viscosity. However, with an increase in shear rate, the external mechanical impact led to the dispersion and contraction of the network-like particles of γ-PGA and FG within the emulsion. As a result, the reduced entanglement between the particles led to shear thinning.

At a constant shear rate, the apparent viscosity of the emulsion gradually increased with an increase in the concentration of γ-PGA. Furthermore, the system exhibited its maximum apparent viscosity when the concentration of γ-PGA reached 0.1%. This observation implied that both γ-PGA and FG, being biomacromolecules, had a partial flocculation effect, influencing the apparent viscosity. Previous studies examined the impact of maltodextrin concentration and glucose equivalent on the stability and rheological properties of corn oil-in-water emulsions. It was observed that the apparent viscosity of the emulsions showed a sharp increase as the maltodextrin concentration surpassed the critical flocculation threshold (39). In this work, when the concentration of γ-PGA increased, it might lead to more severe flocculation and consequently result in an increased apparent viscosity.

#### 3.1.2 Frequency sweep

In rheology, the parameters G' and G” are used to describe the elastic (storage) and viscous (loss) components of a material's response to deformation, respectively. These parameters are associated with complex shear modulus. When G' is higher than G”, the rigidity of emulsion is dominant, and emulsion mainly shows solid characteristics. When G” > G', the flexible of gel emulsion is dominant and it mainly shows liquid characteristics.

**Figure 2:**
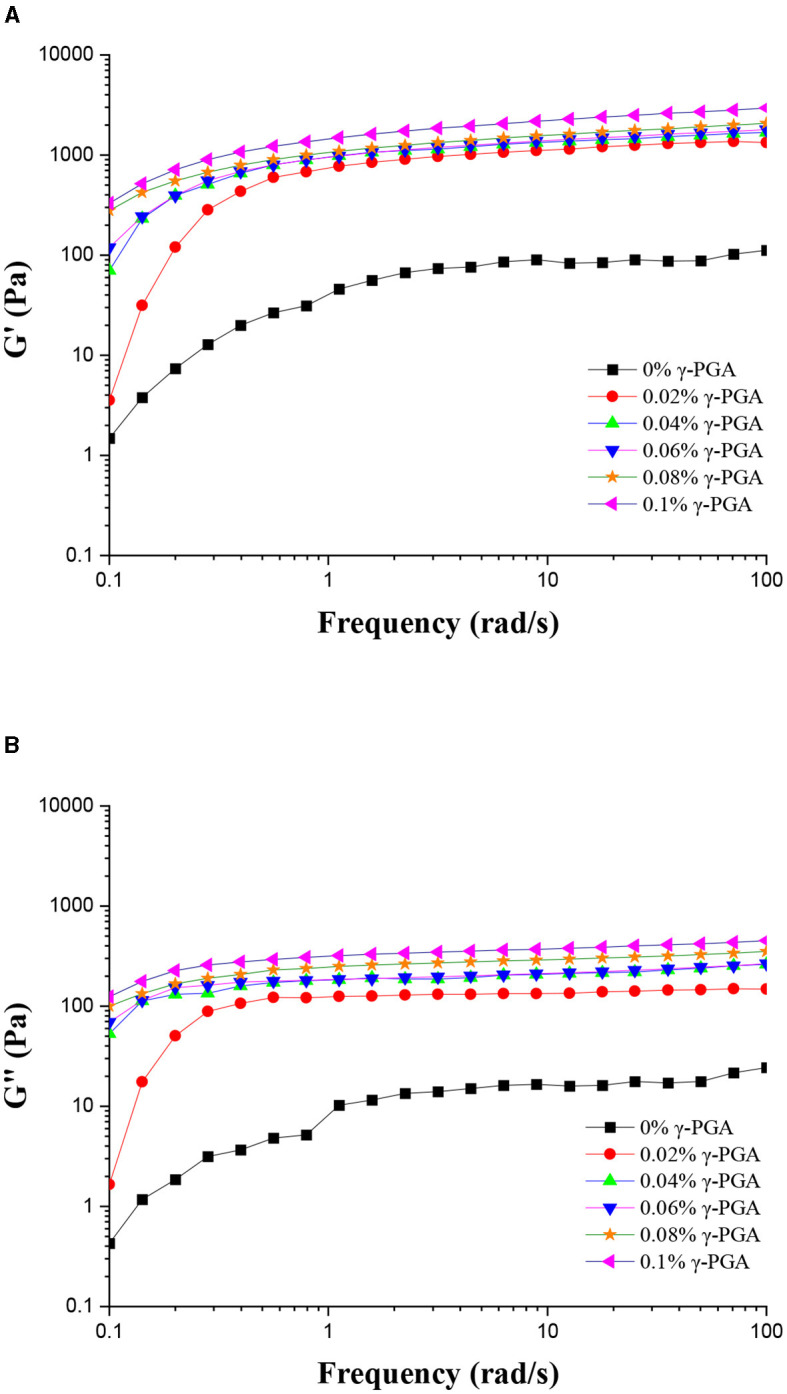
Frequency sweep curves of FG emulsions with different concentrations of γ-PGA: G' **(A)** and G” **(B)**.

When no γ-PGA was added, G' of the emulsion was found to be larger than G”, indicating a dominant elastic behavior. Specifically, the G' value of the emulsion was ~1 order of magnitude higher than the G” value, highlighting the significant difference in stiffness between the two moduli. Other emulsions containing proteins, such as soy protein isolate and whey protein, have also been shown to exhibit analogous characteristics (40). Furthermore, as the frequency increased, the values of both G' and G” increased gradually, establishing a clear positive correlation between frequency and the mechanical properties of the system. In the presence of γ-PGA, the G' value of the emulsion exhibited higher values than the G” value, indicating a more pronounced elastic behavior. Additionally, both the G' and G” values of the emulsion were significantly higher compared to the control group, suggesting an enhanced mechanical response attributed to the addition of γ-PGA. The concentration of γ-PGA directly influenced the values of both G' and G” in a proportional manner, with higher γ-PGA concentrations resulting in higher G' and G” values. Similarly, on the addition of γ-PGA to FG emulsions, both G' and G” values increased as the frequency increased. However, it was noteworthy that the magnitude of this change was comparatively smaller than that observed in the control group, suggesting a moderate effect of γ-PGA addition on the frequency-dependent mechanical properties of FG emulsions. At an angular frequency of 100 rad/s and addition of γ-PGA at a concentration of 0.1%, the emulsions demonstrated the highest values for both G' and G”. Moreover, these values were significantly higher than those observed in the emulsions in which γ-PGA was not added (Figure 2). The results implied that the addition of γ-PGA had the potential to enhance the viscoelastic properties of FG emulsions, possibly due to its ability to strengthen the interconnected network structure within the emulsion. This suggests that γ-PGA could play a crucial role in improving the overall mechanical properties and stability of the emulsion system. At γ-PGA concentrations exceeding 0.04%, both G' and G” values began to decrease. Conversely, when γ-PGA concentrations were below 0.04%, the values of G' and G” exhibited substantial variations with frequency. These findings suggested that a higher concentration of γ-PGA in FG emulsions had reached a level of structural strength, while emulsions with a lower concentration of γ-PGA were more susceptible to frequency-induced changes, indicating a comparatively weaker structural stability.

#### 3.1.3 Temperature scan

**Figure 3:**
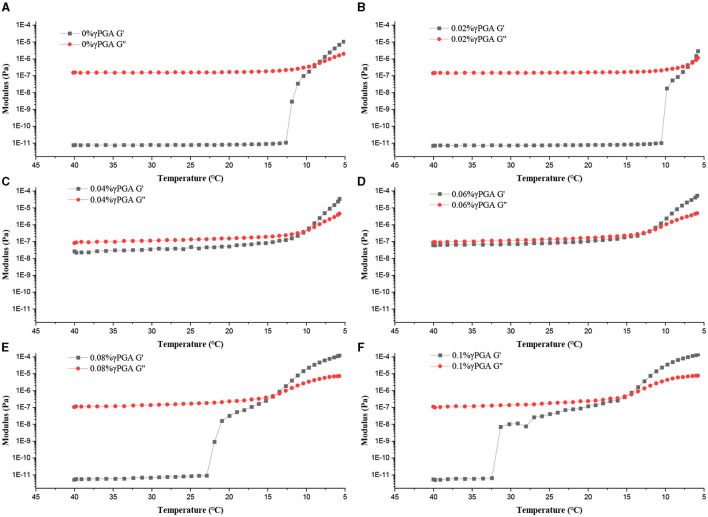
Temperature scanning curves of FG emulsions with different concentrations of γ-PGA (0, 0.02, 0.04, 0.06, 0.08, 0.1% from **(A)**–**(F)**, respectively).

According to Figure 3, the G” values of all the samples were higher values than the G' values at an elevated temperature, indicating a predominant liquid-like behavior at those conditions. Initially, as the temperature decreased, the values of G' and G” showed minimal changes. However, at specific temperature thresholds, a significant and rapid increase was observed in both moduli. Eventually, upon reaching the gelation temperature, the G' value was equal to G” value, resulting in the transition of the sample into a solid-like state. We observed that the higher concentration of γ-PGA, the higher gelation temperature of the emulsion gel. When the concentration of γ-PGA was 0.1%, the gelation temperature was the highest, rising from the initial 8.63°C to 15.05 °C. This might be because γ-PGA successfully modified the FG skeleton (41). With the decrease in temperature, the FG chains transform from a random coiling to a triple helix structure, thereby promoting the formation of a gel network. FG and γ-PGA formed a complex through non-covalent interactions, such as electrostatic interaction, hydrogen bonding, and van der Waals forces. When the concentration of γ-PGA increased, the interaction between FG and γ-PGA is strengthened, resulting in an increase in gelation temperature. (29). The interaction between γ-PGA and FG leads to the formation of an emulsion gel framework, with the oil droplets being encapsulated within this framework. As the gelation temperature of the aqueous phase in the emulsion gel increased, the gelation temperature of the entire system also rose in parallel.

### 3.2 Stability analysis

#### 3.2.1 Storage stability

Emulsions must be stable during storage to maintain their quality. If emulsions were unstable, droplets could float to the surface, became cohesive, and eventually separate. Therefore, maintaining the stability of the emulsion storage is critical for preserving the quality over time.

**Figure 4:**
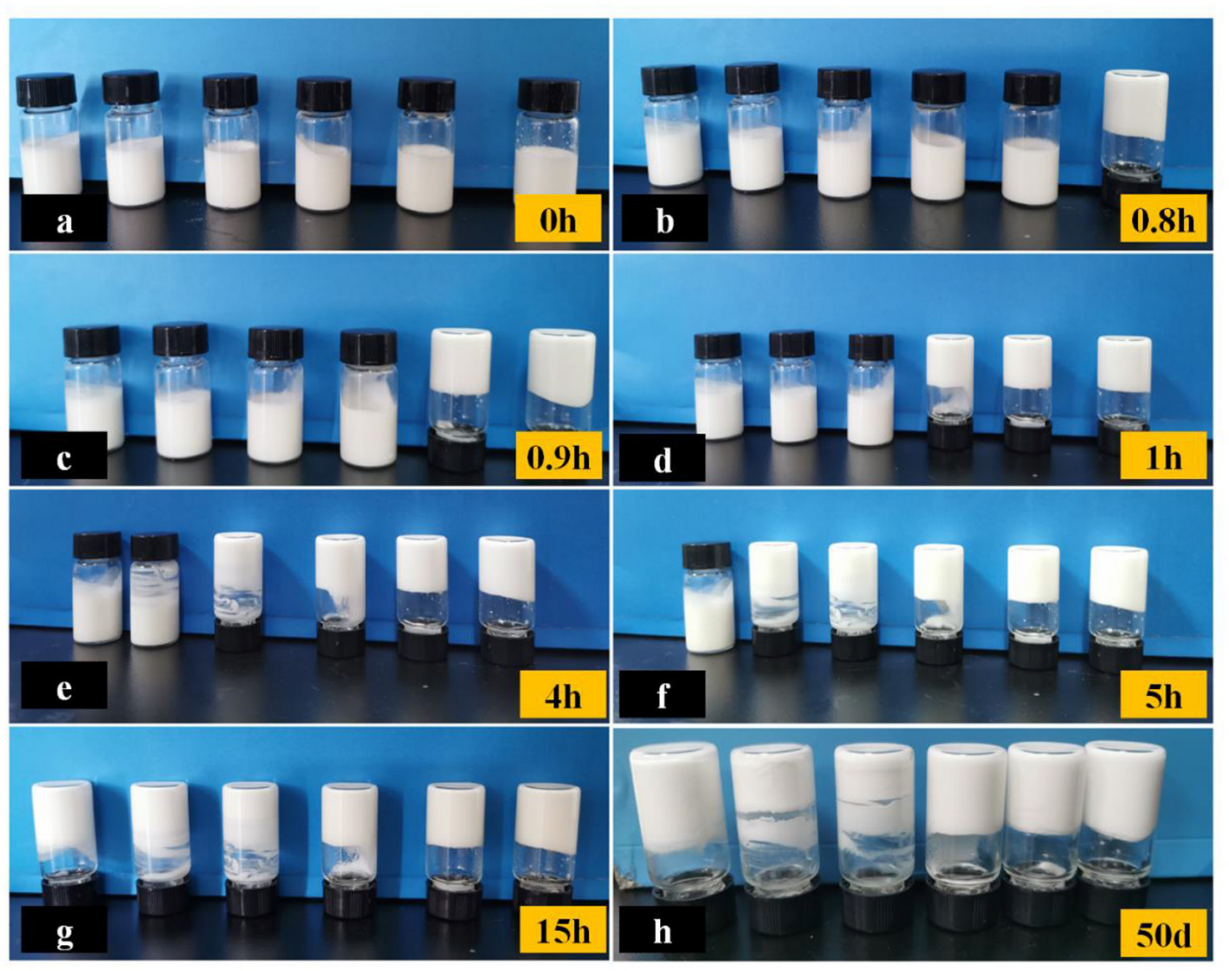
Storage stability of FG emulsions with different γ-PGA concentrations (0, 0.02, 0.04, 0.06, 0.08, 0.1% from left to right, respectively) at different time **(a–h)**.

The storage stability can be assessed based on the gelling time. Initially, all emulsions were homogeneous and fluid (Figure 4a). As depicted in Figures 4b–g, a higher concentration of added γ-PGA resulted in a shorter time for the solidification of the emulsion. Notably, the emulsion with a concentration of 0.1% γ-PGA solidified first at 0.8 h (Figure 4b). In this state, the formed emulsion gel exhibited non-flowing behavior even when inverted. After 15 h, all emulsions had completely solidified (Figure 4g).

After the emulsion gels were formed, they remained at the uniform state for a long time. As shown in Figure 4h, even after 50 days of storage, there was no obvious change in each emulsion gel without stratification. The composite structure of the emulsion gel can be described as a hybrid network consisting of cross-linked biopolymer molecules and partially aggregated droplets (42). The biomacromolecules in the aqueous phase were FG and γ-PGA. On the one hand, the gel network was strengthened by the non-covalent interactions between FG and γ-PGA, as well as between FG and FG, resulting in the effective entrapment of oil droplets. As a consequence, the gelling time was reduced. A faster gelation process prevented the droplets from separating from the water phase and thus extended the storage time. On the other hand, an increase in the viscosity of γ-PGA led to a decrease in the flow rate of droplets. This decrease in flow rate subsequently reduced their collision and aggregation, thereby maintaining the stability of the system. Pectin, being a biological macromolecule, also exhibited good stability in emulsions when high-viscosity pectin was incorporated (43).

#### 3.2.2 Freeze–thaw stability

**Figure 5:**
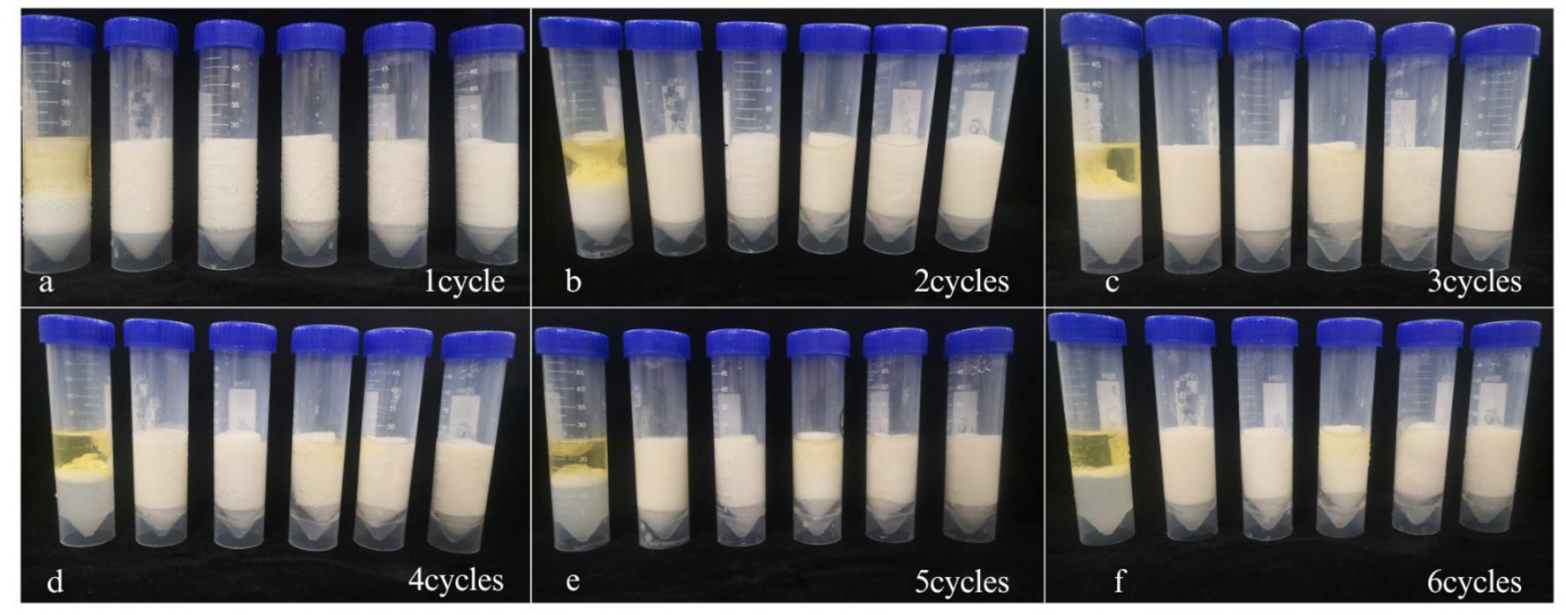
Freeze-thaw stability of FG emulsion gels with different concentrations of γ-PGA (0, 0.02, 0.04, 0.06, 0.08, 0.1% from left to right, respectively) after different cycles **(a–f)**.

Freeze–thaw stability is an important property for emulsions that needed to be frozen prior to consumption, such as sauces and some beverages. When the emulsions were frozen, the water and oil phases could crystallize, thereby impacting the stability and properties of the resulting frozen emulsions. Thawing frozen emulsions could lead to partial breakdown, causing oil separation known as oiling off. (44). Emulsion gels of different degrees of instability were observed when subjected to freeze–thaw cycles ranging from −20°C to 20°C, mainly in the form of phase separation. After the freeze–thaw treatment, the emulsion gels exhibited demulsification. Shown in Figure 5a, one freeze–thaw cycle resulted in stratification of FG emulsion gel without γ-PGA, while two cycles led to significant oil precipitation. More than two freeze–thaw cycles completely separated the oil phase and water phase of the emulsion gel. It indicated that the FG emulsion gel not modified by γ-PGA had poor stability. By contrast, on the addition of γ-PGA, the emulsion gel did not undergo obvious stratification even after several freeze–thaw cycles (Figures 5b–f). The results showed that the addition of γ-PGA provides a certain antifreezing effect. The emulsion gel was unstable when it thawed probably because droplets were forced together when water and oil phase ice crystals formed. It has been reported to increase the viscosity of non-frozen aqueous solutions, form a protective coating, and prevent the polymerization of oil droplets in emulsions (45). Studies have demonstrated that γ-PGA significantly improves the freeze resistance of yeast cells and sweet dough by exerting a stabilizing effect on water molecules, thereby preventing water crystallization and effectively inhibiting freezing (46). γ-PGA had been observed to safeguard the quality of grass carp surimi during freezing storage by reducing ice crystal formation, consequently minimizing cellular and tissue damage (47). The improved antifreeze effect of FG emulsion with the addition of γ-PGA could be attributed to multiple factors. Firstly, γ-PGA demonstrated the capability to delay the crystallization of the water phase, thus effectively preventing the formation of ice crystals. Moreover, the strong interaction and formation of a dense lattice structure between γ-PGA and FG contributed to their resistance against separation due to external forces, thereby preventing dehydration. Additionally, the inclusion of γ-PGA increased the viscosity of the unfrozen water phase, leading to a higher proportion of unfrozen water within the emulsion gel. This increased viscosity also resulted in a reduction in droplet–droplet collisions, subsequently reducing droplet aggregation. Collectively, these mechanisms merged to enhance the antifreeze effect of FG emulsion when supplemented with γ-PGA. When the freeze–thaw cycle count exceeded three, the magnitude of external forces increased, eventually surpassing the critical threshold of the system. As a result, the emulsion gel containing high concentration of γ-PGA also began to exhibit an oil–water separation phenomenon.

### 3.3 Gel characterization

**Figure 6:**
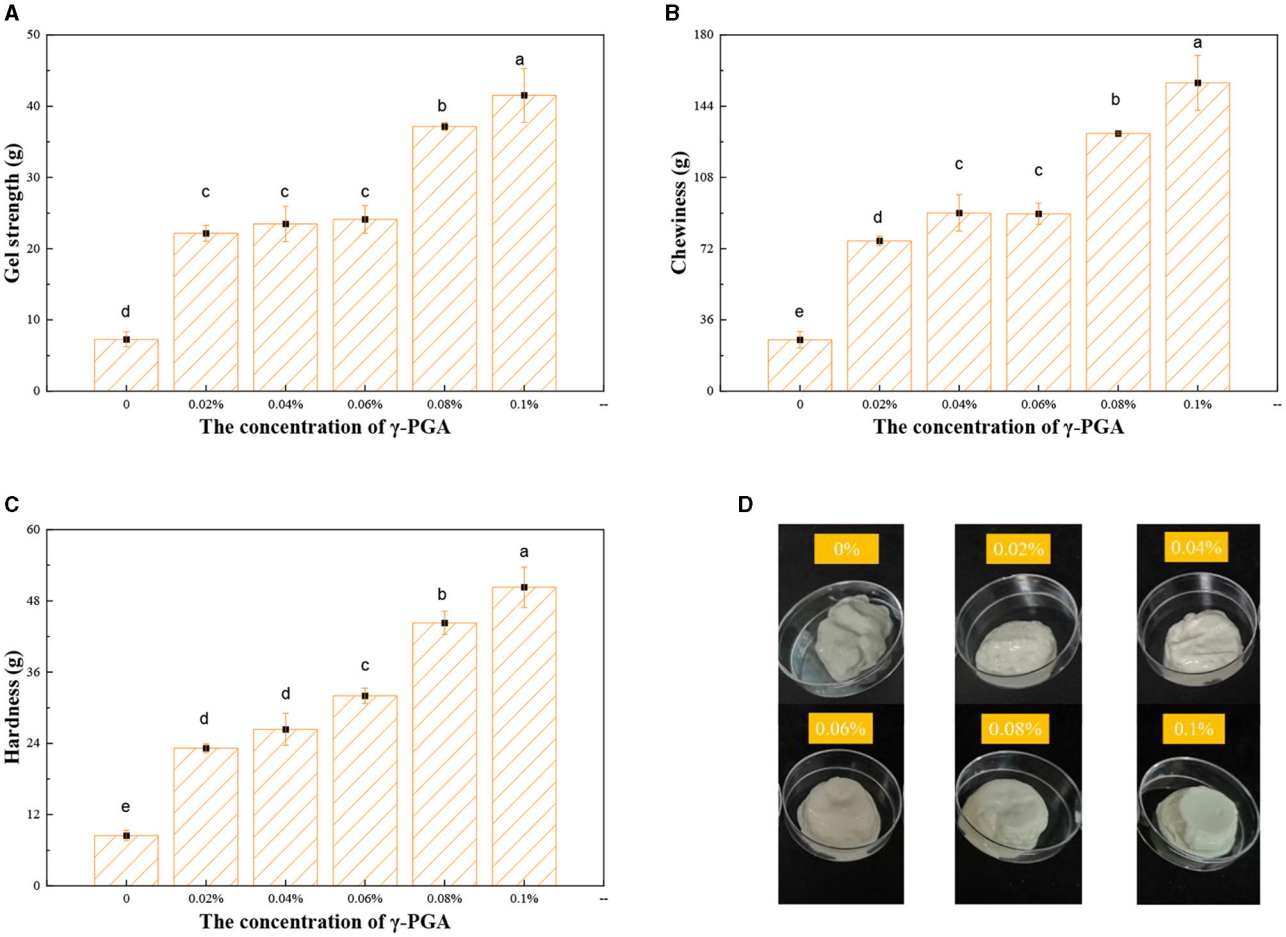
Gel strength **(A)**, hardness **(B)**, and chewiness **(C)** of FG emulsion gels with different concentrations of γ-PGA and visual presentation of emulsion gels **(D)**.

As shown in Figure 6D, all samples were cold-treated to form emulsion gels. FG as the aqueous phase of emulsion formed a gel of certain strength together with γ-PGA as the skeleton of emulsion gel at low temperature. The emulsion gel without γ-PGA had a gel strength of only 7 g (Figure 6A). However, when the γ-PGA concentration reached 0.1%, the gel strength of emulsion gels reached 41.51 g, which is a 4.7-fold increase than the control group. The gel strength of emulsion gels increased with the increase of γ-PGA concentration. It might be due to the fact that FG and γ-PGA could form complexes through non-covalent interactions and introduce γ-PGA into the FG molecules. Because γ-PGA was negatively charged in the system and could form polymers through electrostatic interactions with positively charged FG, which also enhanced the gel network structure. The viscosity of γ-PGA also helped to provide a thicker texture, helping in better enveloping of the oil droplets (48). By confining the oil droplets to the hydrogel network, hydrogel networks were formed to fix the droplets within the gel structure, thus preventing flocculation and coalescence (49).

Chewiness is a characteristic of food texture that encompasses both the perception of taste and the level of difficulty encountered during mastication in the oral cavity. The chewiness of the emulsion gel demonstrates a significant increase in tandem with the increase in the concentration of γ-PGA (Figure 6B). This phenomenon could be attributed to the enhanced encapsulation of oil droplets by FG, facilitated by higher γ-PGA concentrations. Consequently, the overall chewiness was improved. Additionally, the combination of elevated concentrations of both γ-PGA and FG fosters the development of a more organized and densely packed protein network structure. This structural arrangement offers increased resistance against external pressure, thus contributing to the superior chewing properties of the emulsion gel.

The hardness parameter provides a reliable measure of softness by quantifying the peak force exerted during testing. Notably, the variation in hardness closely mirrors that of gel strength, suggesting a robust correlation between these two factors. Specifically, the study revealed that at a γ-PGA concentration of 0.1%, the emulsion gel attained a maximum hardness value of 50.36 g, which was nearly five times higher compared to the gel that did not contain γ-PGA (Figure 6C). This significant disparity highlights the pronounced influence of γ-PGA concentration on the mechanical properties of the emulsion gel.

### 3.4 Analysis of water-holding capacity

**Figure 7:**
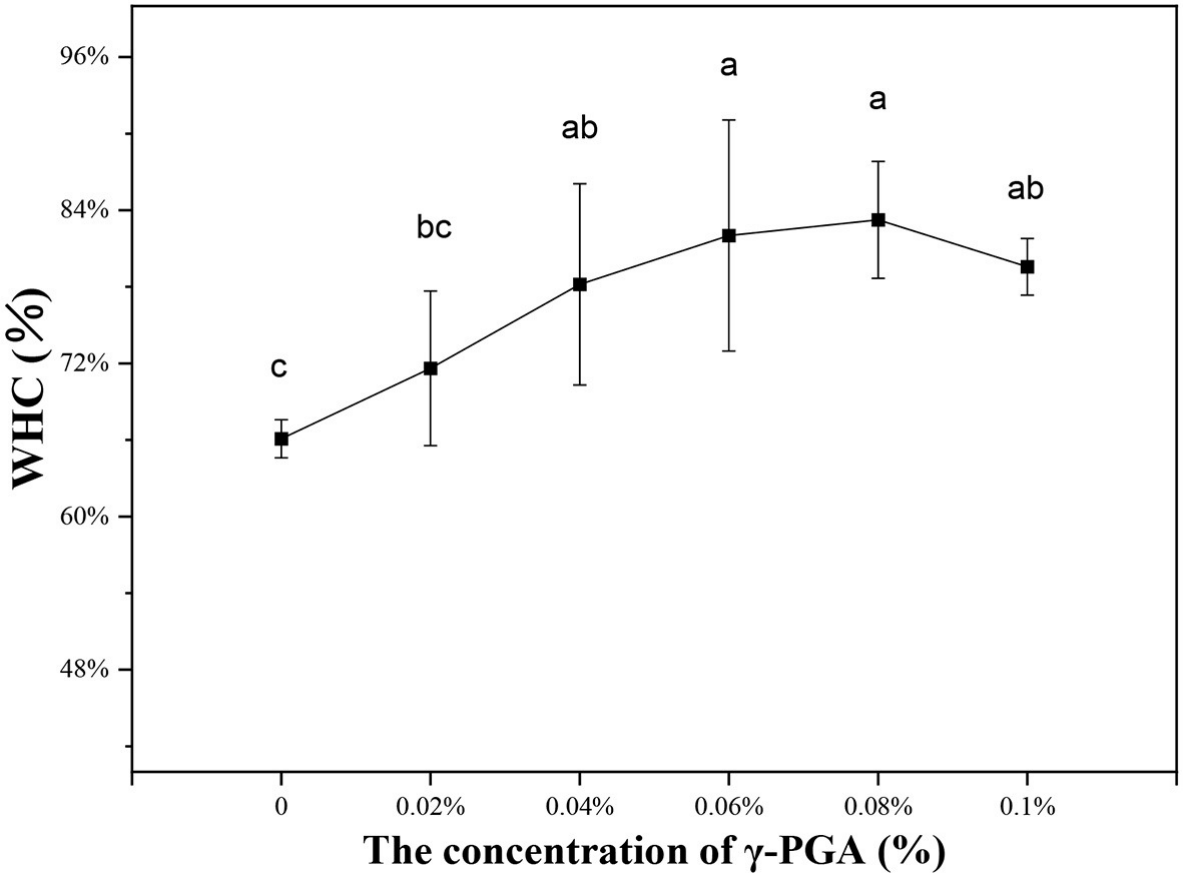
Water-holding capacity of FG emulsion gels with γ-PGA in various concentrations.

The water-holding properties of emulsion gel affected its quality attributes. Moreover, it's also related to the stability of the system (50). A small amount of water was observed at the bottom of the centrifuge tubes of all the samples. Less water was released after centrifugation and the WHC increased when γ-PGA concentration was increased from 0% to 0.1%. As shown in Figure 7, the WHC of emulsion gel without γ-PGA was only 66.1% and it increased significantly to 83.25% when the concentration of the added γ-PGA was 0.08%. The results of this experiment were similar to observations in previous studies. Yu et al. found that the WHC of yogurt increased with the amount of γ-PGA (51). Xie et al. found that with the addition of γ-PGA, the WHC of wheat gluten increased (52). The WHC of the emulsion gel was closely related to the structure and strength of the gel-like network (53). The WHC can significantly affect the gel's properties. For instance, increasing the WHC of a gel can increase its firmness, elasticity, viscosity, and stability (54). Furthermore, gels with high water-holding capacity tend to have enhanced textural properties, which could improve their functional abilities in various food and non-food applications (55). Going by the experimental results of gel strength, it was also proved that WHC might be related to the network structure of emulsion gel. When γ-PGA concentration was low or it was not added, the binding ability of free water was poor due to the weak structure of emulsion gel and large pore size. The increase of WHC in high-concentration γ-PGA–FG emulsion gel might be due to the enhancement of gel network composed of FG and γ-PGA. When the γ-PGA concentration increased, the pore size of this network decreased, thus enhancing the capillary force that holds water in the hydrogel. In addition to water, the oil phase was also more tightly wrapped by the network, which made the emulsion gel more stable. This was also consistent with the other stability experiments highlighted above. Besides, the increase of WHC might be also related to the high hydrophilic function and water absorption capacity of γ-PGA. The addition of Persian gum, which also has high hydrophilicity, has been reported to increase WHC (56). The more water bound to it, the stronger network formed with FG emulsion, and the less water spilt after centrifugation.

### 3.5 Droplet characterization

#### 3.5.1 Confocal laser scanning microscopy

**Figure 8:**
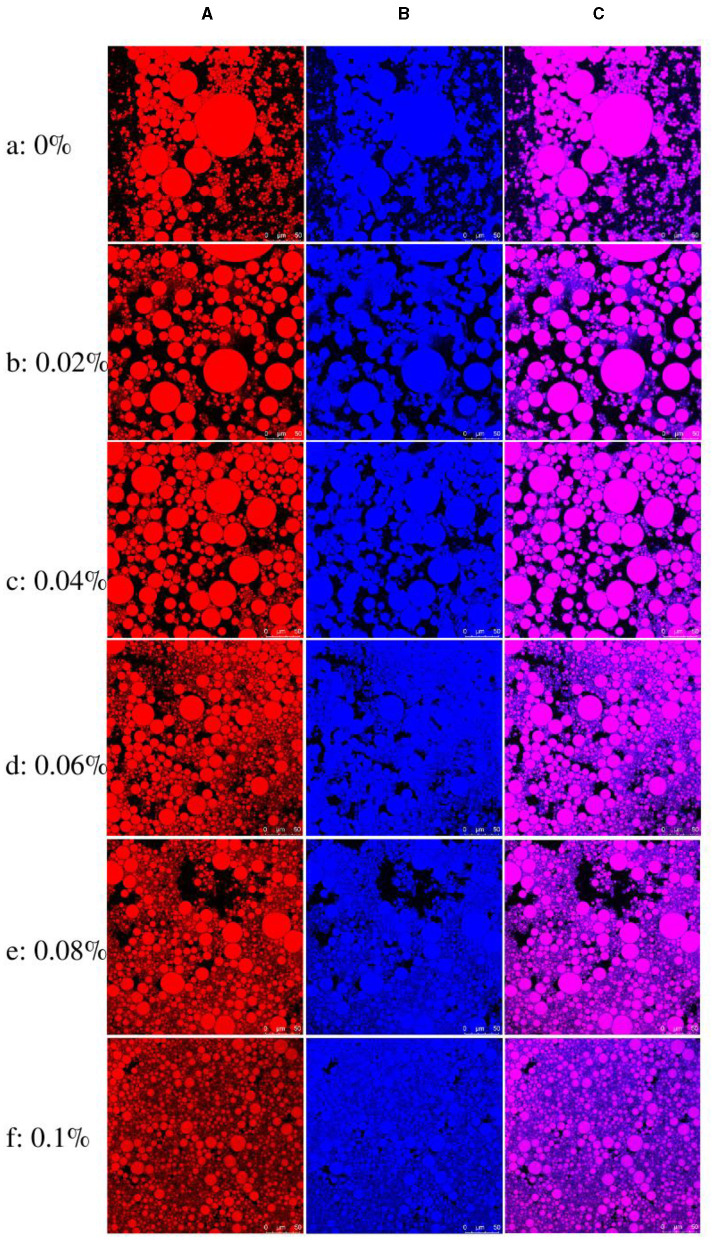
CLSM images of FG emulsion gels with different concentrations of γ-PGA: **(A)** stained using Nile red; **(B)** stained using Nile blue; and **(C)** combined image of panels **(A, B)**.

Confocal laser scanning microscopy is well suited to the characterization of emulsion gel systems, from which particle or droplet size distributions can be obtained (57). After dying, we could see the components of the emulsion gel more clearly. Nile red was used to stain oil-soluble substances and Nile blue to stain water-soluble substances. To observe the microstructure of the emulsion gel, corn oil was stained with Nile red dye, and FG and γ-PGA were stained with Nile blue dye (Figures 8A, B).

Figure 8C provides visual evidence demonstrating the characteristics of the emulsion gel observed in the control group. Notably, the control group exhibited a high degree of variability in particle size, coupled with an uneven distribution. Local aggregation of particles was also evident within the gel structure.

Conversely, with the increase in γ-PGA concentration, a significant reduction in droplet size was observed. This effect was best exemplified in Figure 8Cf, which represents the emulsion gel at a concentration of 0.1% γ-PGA. Here, the particle size reached its smallest value, displaying a more uniform distribution across the gel matrix. Additionally, the presence of FG and γ-PGA can be prominently observed, dispersed evenly throughout the background. These components contributed to the formation of a well-defined gel skeleton within the emulsion gel structure.

There might be two reasons for the decrease of droplet size with the increase of γ-PGA concentration. One was that the higher viscosity of the aqueous phase might result in stronger shear forces during homogenization, which led to the formation of smaller oil droplets. The second was that the presence of a strong hydrogel network around the oil droplets might have inhibited their aggregation. This was also consistent with the result that high concentration of γ-PGA enhanced the gel strength of emulsion gels.

#### 3.5.2 Particle size

**Figure 9:**
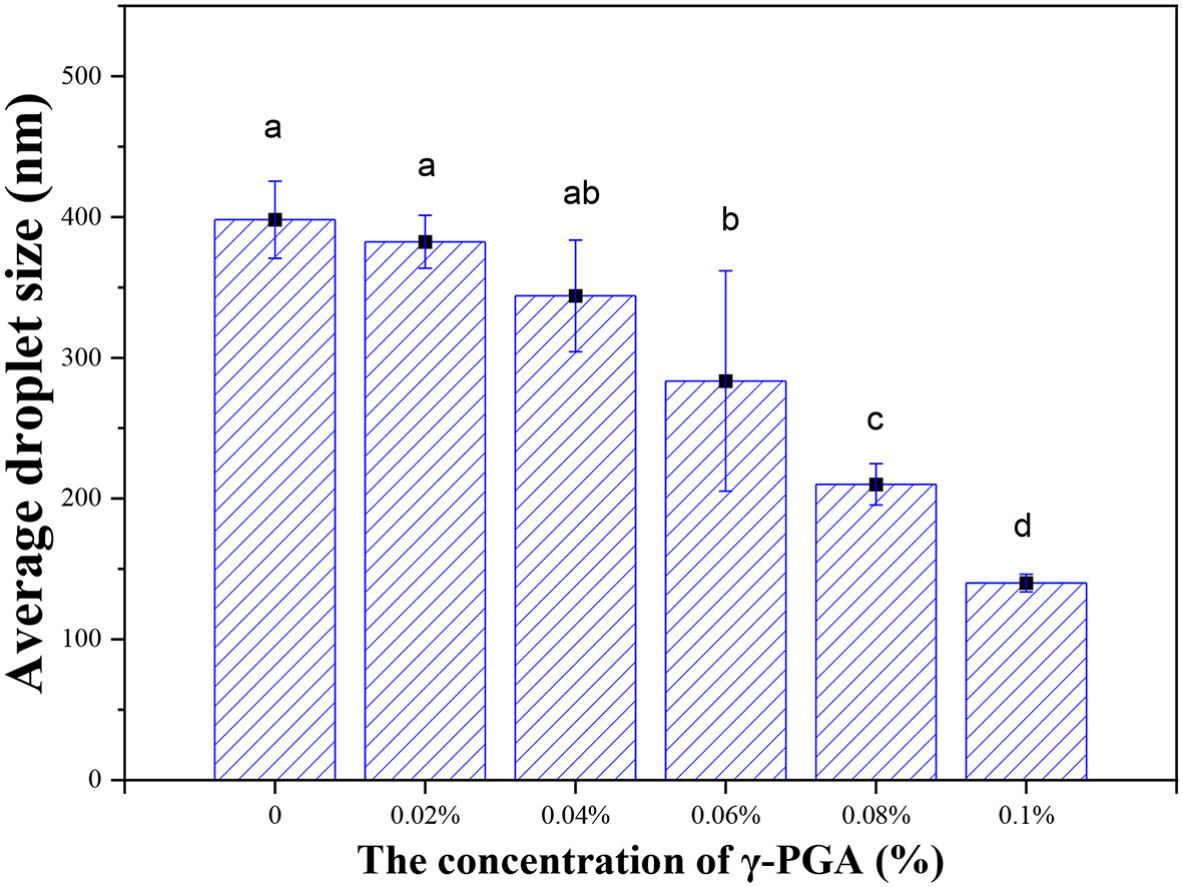
Average droplet size of FG emulsion gels with different concentrations of γ-PGA.

In Figure 9, the average particle size of FG emulsion without γ-PGA was found to be the largest, measuring ~398 nm. However, as the concentration of γ-PGA increased, there was a gradual decrease in the particle size of the emulsion. Under the most optimized conditions, the average diameter of FG emulsion droplets was determined to be 139.99 ± 24.7 nm at a γ-PGA concentration of 0.1%. This reduction in particle size was remarkable, as it indicated that the particle size of the emulsion with γ-PGA was only one-third that of the FG emulsion without γ-PGA.

The observed trend in the reduction of particle size was further supported by CLSM findings. Thus, a consistency between the measured particle size and the visual observations was observed. Such a reduction in particle size can be attributed to the elevated viscosity conferred by γ-PGA. This increased viscosity restricts droplet collisions, subsequently delaying the process of droplet agglomeration. It is well known that smaller droplets contribute to enhanced system stability, reinforcing the findings obtained in previous stability analyses.

Overall, these results reaffirm the role of γ-PGA in reducing the particle size of FG emulsion, leading to improved stability and suggesting its potential as a beneficial additive in emulsion systems.

## 4 Conclusions

This research found that γ-PGA acted as an effective stabilizer for oil-in-water FG emulsion gel. γ-PGA increased the apparent viscosity of the FG emulsion gel and the viscosity of the modified FG was the highest when the concentration of γ-PGA was 0.1%. Temperature scanning of emulsions indicated that the higher the concentration of γ-PGA, the higher the gelation temperature of the emulsion. When the γ-PGA concentration was 0.1%, the gelation temperature was the highest, rising from the initial 8.63°C to 15.05 °C, which is a nearly 2-fold increase. The freeze–thaw stability experiment showed that the FG emulsions modified by γ-PGA was better able to withstand repeated freeze–thaw cycles. γ-PGA increased the stability of the FG emulsion gel. The analysis of the gelling properties demonstrated a notable increase in gel strength as the concentration of γ-PGA increased. Specifically, the emulsion gel with a γ-PGA concentration of 0.1% exhibited a 4.7-fold increase in gel strength compared to the control group. The WHC of emulsion gel containing only FG in aqueous phase was only 66.1%, which was significantly increased to 83.25% after the addition of 0.08% of γ-PGA. The CLSM results showed that γ-PGA concentrations had significant influence on the particle size of the emulsion gel, with smaller droplets being observed with the increase of γ-PGA concentrations. When the γ-PGA concentration reached 0.1%, the observed droplets were the smallest and most evenly distributed. The results of this study have clearly demonstrated the ability of γ-PGA to effectively improve the properties of FG emulsion gel for its application in food, medicine, cosmetics, and other industries.

## Data availability statement

The original contributions presented in the study are included in the article/supplementary material, further inquiries can be directed to the corresponding authors.

## Author contributions

HX: Writing – original draft, Software, Methodology, Investigation, Conceptualization. X-MS: Writing – review & editing, Funding acquisition. PY: Writing – review & editing, Validation. J-LL: Writing – review & editing, Validation, Formal analysis. Z-ZH: Writing – review & editing, Validation, Methodology, Formal analysis. Z-CT: Writing – review & editing, Supervision, Funding acquisition.
